# Antimicrobial strategies for ureteral stent removal after radical cystectomy: a comparative cohort study

**DOI:** 10.1007/s10096-026-05514-4

**Published:** 2026-04-30

**Authors:** Maurin Helen Mangold, Jennifer Kazmierczak, Marlis Gerigk, Nicolas Carl, Luisa Vivienne Renner, Maren Juliane Wenk, Maurice Stephan Michel, Karl-Friedrich Kowalewski, Niklas Westhoff

**Affiliations:** 1 Department of Urology and Urosurgery, Medical Faculty Mannheim, University Medical Center Mannheim, Heidelberg University, Mannheim, Germany; 2 German Cancer Research Center (DKFZ) Heidelberg, Division of Intelligent Systems and Robotics in Urology (ISRU), Heidelberg, Germany; 3 Department of Medical Microbiology and Hygiene, Medical Faculty Mannheim, University Medical Center Mannheim, Heidelberg University, Mannheim, Germany

**Keywords:** Radical Cystectomy, Urinary Diversion, Ureteral Stent Removal, Antimicrobial Strategies

## Abstract

**Introduction:**

Patients undergoing radical cystectomy (RC) face a heightened risk of infectious complications around the time of ureteral stent removal. This study aimed to determine whether a culture-guided antimicrobial strategy reduces infectious complications after stent removal compared with an empirical nitrofurantoin regimen.

**Methods:**

We analyzed 200 patients who underwent RC with urinary diversion and ureteral stenting. Patients received either empirical nitrofurantoin or a culture-guided antimicrobial strategy based on pre-removal urine cultures for stent removal. The primary endpoint was the incidence of early systemic urinary tract infection (UTI) within 48 h after stent removal. Secondary endpoints included changes in inflammatory markers, hydronephrosis after stent removal, and UTI-related readmissions within 30 and 90 days.

**Results:**

Early systemic UTIs occurred in 4% of patients receiving empirical nitrofurantoin and 7% in the culture-guided group (OR 0.56, 95% CI 0.12-2.27; *p* = 0.54). UTI-related readmissions at 30 and 90 days were similar between regimens (1% vs. 2%, *p* = 0.99; 5% vs. 9%, *p* = 0.41). An early leukocyte rise was associated with early systemic UTI after stent removal (2.65 vs. 0.51 × 10⁹/L; *p* = 0.048). Pre-removal urine cultures frequently yielded non-classical uropathogens consistent with stent-associated colonization, potentially limiting the clinical utility of routine culture-guided escalation. The culture-guided strategy did not reduce early infectious complications despite the use of broader-spectrum agents.

**Conclusion:**

Empirical nitrofurantoin and a culture-guided antimicrobial strategy showed similar rates of early systemic UTIs and UTI-related readmissions after ureteral stent removal in patients undergoing RC. Routine culture-guided escalation may therefore offer limited clinical benefit while increasing antimicrobial exposure. Prospective multicenter trials are warranted to define optimal procedure-specific strategies.

**Supplementary Information:**

The online version contains supplementary material available at 10.1007/s10096-026-05514-4.

## Introduction

Radical cystectomy (RC) with urinary diversion (UD) carries a substantial risk of infectious complications during the early postoperative period [1]. Despite standardized perioperative antibiotic prophylaxis, urinary tract infections (UTIs) occur in 10-36% of patients within 90 days after RC and have even been linked to poorer overall survival [2]. As both ileal conduit (IC) and orthotopic neobladder (NB) reconstructions typically require ureteral stents to support initial anastomotic healing, stent-related factors contribute considerably to this risk of infection. Ureteral stents rapidly develop biofilm and become colonized in most patients, with colonization reported in nearly all permanently and approximately 70% of temporarily stented patients [3]. This substantial colonization burden provides a persistent microbial reservoir, and the 2025 EAU Guidelines recognize ureteral stenting as an independent risk factor for both localized and systemic UTI [3]. Although asymptomatic bacteriuria (ABU) in patients with indwelling ureteral stents should generally not be treated, the period surrounding ureteral stent removal after RC represents a clinically vulnerable phase and has been associated with an increased risk of subsequent UTIs [4, 5]. This highlights the need to critically evaluate antimicrobial strategies specifically for ureteral stent removal after RC. Despite its clinical relevance, no current guideline provides recommendations on whether antimicrobial prophylaxis is necessary for ureteral stent removal after RC. While the overall quality of evidence remains limited, several studies suggest that the use of antimicrobial prophylaxis, compared with no prophylaxis, may reduce infection-related complications during ureteral stent removal: A recent systematic review reported lower median rates of positive blood cultures when any antimicrobial prophylaxis was used before stent removal [6]. Reported strategies included culture-guided single-dose intravenous regimens as well as empirical single-dose or short-course (≤ 2-day) antibiotic prophylaxis using third-generation cephalosporins, aminoglycosides, fluoroquinolones, or trimethoprim-sulfamethoxazole. However, the optimal agent, timing, and duration remain undefined, leaving clinicians without evidence-based guidance.

To inform evidence-based management, we conducted a comparative evaluation of two antibiotic strategies for ureteral stent removal following RC used in routine practice at a high-volume RC center: **(1)** an empirical prophylactic regimen consisting of nitrofurantoin 100 mg once daily from postoperative day (POD) 1 until stent removal and **(2)** a culture-guided antibiotic approach based on standardized urine cultures. We hypothesized that culture-guided antimicrobial therapy would be superior to empirical nitrofurantoin in reducing infectious complications after stent removal and might better support rational antimicrobial decision-making in this high-risk population.

## Patients and methods

We adhered to the STROBE statement (**Supplementary Table ****1**).

### Study design

**Fig. 1:**
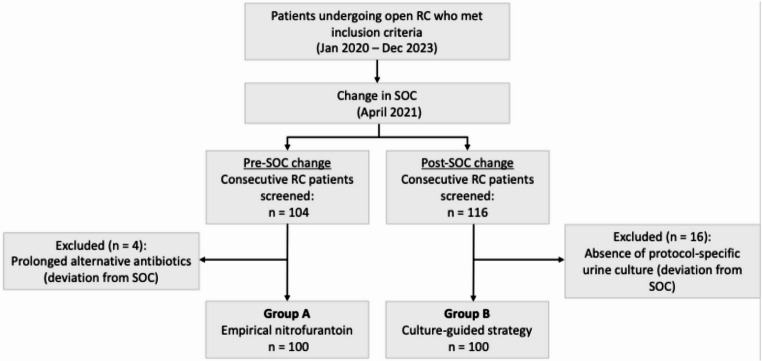
Study flow diagram. In this exploratory retrospective cohort study, the first 100 consecutive eligible patients adhering to the respective SOC regimen before and after the institutional change in standard of care (from empirical nitrofurantoin to a culture-guided antimicrobial strategy) in April 2021 were included for analysis. Inclusion criteria are detailed in the Methods section. RC, radical cystectomy; SOC, standard of care

We conducted a single-center retrospective cohort study including 200 patients aged ≥ 18 years who underwent open RC with ureteral stenting and UD (ileal conduit, orthotopic neobladder, or cutaneous pouch) for muscle-invasive bladder cancer (MIBC) or high-risk non-muscle-invasive bladder cancer (NMIBC) between January 2020 and December 2023. Exclusion criteria were age < 18 years, RC for non-oncological indications, UD other than ileal conduit, orthotopic neobladder, or cutaneous pouch (e.g. ureterocutaneostomy), no UD, or absence of ureteral stenting during RC (Fig. 1). Among all patients, 151 received standard intraoperative single-shot piperacillin/tazobactam prophylaxis due to negative preoperative urine cultures, according to the institutional protocol developed with the in-house microbiology department based on local pathogen distribution and resistance patterns. The remaining 49 patients had significant preoperative bacteriuria: 23 received piperacillin/tazobactam starting 24 h before RC, and 26 had already initiated appropriate culture-directed antimicrobial therapy in the outpatient setting (≥ 24 h before surgery). In both subgroups, the respective antimicrobial regimen was continued for 48 h after RC and was not routinely prolonged until ureteral stent removal. The timing of ureteral stent removal was at the discretion of the operating surgeon, typically between POD 10 and 16. Irrespective of UD type, POD 10 was the earliest possible day for stent removal. Perioperative management followed an institutional enhanced recovery after surgery (ERAS) protocol for open RC. Renal ultrasound and routine laboratory tests were obtained within 24 h before and again within 24 h after stent removal as part of a standardized institutional protocol.

### Institutional antimicrobial strategy and change of standard of care

The empirical regimen was selected in collaboration with the institutional microbiology department, considering local pathogen distribution, resistance patterns, and tolerability. Following a few complicated UTI episodes temporally associated with ureteral stent removal, the institutional standard of care (SOC) was revised in April 2021 towards a culture-guided approach, aiming to enable a more tailored, susceptibility-aligned antimicrobial strategy at the time of ureteral stent removal.

### Sample size and patient selection

Existing literature on antimicrobial strategies at the time of ureteral stent removal after RC is highly heterogeneous, reflecting rapidly evolving antimicrobial strategies and substantial inter-institutional variation in stewardship policies. Against this background, we adopted a pragmatic, exploratory study design. We included the first eligible 100 consecutive RC patients treated under each antimicrobial strategy before and after the change of SOC in April 2021 at our center, resulting in two equally sized cohorts. This approach was chosen to reflect routine clinical practice and to generate hypotheses regarding potential associations between antimicrobial strategies and infection-related outcomes, rather than to detect small between-group differences with confirmatory statistical power:

- Empirical nitrofurantoin group (n = 100): Patients received oral nitrofurantoin 100 mg once daily from POD 1 until ureteral stent removal.
- Culture-guided group (n = 100): Patients underwent a standardized urine culture after RC, typically on POD 6. Urine samples were collected from the lumen of the ureteral stents prior to stent removal and processed using standard quantitative culture methods. If a pathogen was detected, targeted antimicrobial therapy was initiated at least 24 h before stent removal and continued for at least 48 h thereafter. In the absence of detectable pathogens, single-dose fosfomycin prophylaxis was administered on the morning preceding ureteral stent removal.

Patients who deviated from the respective SOC regimen, such as those receiving prolonged alternative antibiotics in the empirical nitrofurantoin group (*n* = 4) or those lacking a stent-removal-specific urine culture in the culture-guided group (*n* = 16), e.g. due to broad-spectrum antimicrobial coverage during a complicated postoperative RC course, were excluded from analysis (Fig. 1).

### Primary objective

The primary endpoint was the incidence of early systemic UTIs within 48 h after ureteral stent removal. Systemic urinary tract infection (sUTI) was defined as a clinically diagnosed infection originating from any site of the urinary tract, accompanied by signs or symptoms of systemic infection, such as fever ≥ 38.0 °C, chills, flank pain, or signs of sepsis, in accordance with current EAU guideline definitions [3]. Secondary endpoints included changes in inflammatory markers, specifically C-reactive protein (CRP) and leukocyte count, before and after stent removal, newly diagnosed hydronephrosis after stent removal, and the need for reintervention due to hydronephrosis (e.g. ureteral re-stenting or percutaneous nephrostomy). In addition, systemic UTI-related readmissions within 30 and 90 days after RC were evaluated as a secondary endpoint.

### Data collection and outcomes

Baseline characteristics including age, sex, Body Mass Index (BMI), Charlson Comorbidity Index (CCI) [7], American Society of Anesthesiologists (ASA) score [8], neoadjuvant chemotherapy and type of UD were extracted from the electronic health record (EHR). Postoperative variables including antibiotic administration, fever episodes, hydronephrosis, laboratory changes and institutional readmissions were likewise retrieved from the EHR. The study was approved by the institutional review board (reference number 2025-829-AF 11) and conducted in accordance with the Declaration of Helsinki. The requirement for written informed consent was omitted due to the retrospective nature of the study.

### Statistical analysis

For descriptive statistics, continuous variables were reported as mean and standard deviation (SD) and categorical variables as counts and percentages. To assess baseline comparability between the empirical nitrofurantoin group and the culture-guided group, continuous variables were compared using the Mann-Whitney U test, chosen a priori given the sample size and expected non-normal distributions. Formal normality testing was not performed [9]. Categorical variables were compared using Fisher’s exact test throughout, to ensure robustness regardless of cell counts. These tests were performed to inform whether propensity score matching (PSM) would be required. As only minimal and clinically non-relevant differences were observed, PSM was omitted. For the primary endpoint of early systemic UTI, group differences were analyzed using Fisher’s exact test, given the expected low event frequency, and results were expressed as odds ratios (ORs) with corresponding 95% confidence intervals (CIs). Secondary endpoints, including rates of new-onset hydronephrosis, reintervention, and infection-related readmissions at 30 and 90 days, were similarly compared using Fisher’s exact test with ORs and 95% CIs reported where applicable. Changes in inflammatory markers (CRP and leukocyte count) from baseline to post-stent removal were analyzed using the Mann-Whitney U test and reported as mean with standard deviation. P-values of < 0.05 were considered statistically significant. All statistical analyses were performed using R software (version 4.3.1; R Foundation for Statistical Computing, Vienna, Austria).

## Results

A total of 200 patients undergoing open RC were included in the analysis, allocated evenly to an empirical nitrofurantoin group and a culture-guided group. Baseline demographic and clinical characteristics, including age, sex, BMI, ASA score, CCI, type of UD, presence of diabetes, malignant pre-existing illness, and major postoperative complications (Clavien-Dindo grade ≥ IIIB) after RC, were comparable between groups (all *p* > 0.05; Table 1). Mean ureteral stent dwell time was 10.3 days (SD 1.4) in the empirical nitrofurantoin group and 12.0 days (SD 1.6) in the culture-guided group.

**Table 1:** Baseline patient characteristics of the study cohort stratified by antibiotic strategy. Continuous variables are presented as mean ± standard deviation (SD) and categorical variables as counts (percentages). BMI = body mass index; CCI = Charlson Comorbidity Index; ASA = American Society of Anesthesiologists. CDC ≥IIIb indicates major complications according to the Clavien-Dindo classification (grade IIIb or higher). Percentages are calculated from available data and may not sum to 100% due to missing values, which were not imputed.

	Empirical Nitrofurantoin Group n=100	Culture-Guided Group n=100	P-values
**Age in years** (mean (SD))	65.9 (8.82)	68.1 (10.52)	0.107
**BMI in kg/m²** (mean (SD))	26.9 (4.63)	27.1 (6.03)	0.75
**Total CCI points** (mean (SD))	3.6 (2.23)	3.4 (1.76)	0.48
**Diabetes** (n (%))	16 (16)	22 (22)	0.45
**Malignant preexisting illness **(n (%))	20 (20)	20 (20)	1.00
**Gender** (n (%))			0.28
male	75 (75)	68 (68)	
female	25 (25)	32 (32)	
**Neoadjuvant Chemotherapy** (n (%))	10 (10)	20 (20)	0.07
**Preoperative ASA Score **(n (%))			0.56
<3	61 (61.6)	56 (57.1)	
≥3	38 (38.4)	42 (42.9)	
**Urinary Diversion** (n (%))			0.19
Ileal conduit	43 (43)	55 (55)	
Ileal neobladder	52 (52)	42 (41)	
Pouch	5 (5)	3 (3)	
**CDC ≥ IIIb** (%)	7 (7)	6 (6)	0.77

### Pathogen spectrum in patients with indwelling ureteral stents after RC

Of the 100 patients in the culture-guided group, 17 patients (17%) showed no bacterial growth in the urine culture obtained prior to ureteral stent removal. Twelve patients (12%) demonstrated bacterial or fungal growth only at a non-significant colony count (≤ 10^3^ colony-forming units (CFU)/mL). The remaining 71 patients (71%) exhibited significant (≥ 10⁴ CFU/mL) bacterial or fungal growth, with the corresponding pathogen spectrum detailed in Table 2. Overall, only about one-third of isolates represented classical uropathogens, whereas the majority consisted of likely biofilm-associated organisms or contaminants, with a marked predominance of Candida spp. and coagulase-negative staphylococci (Table 2). Among positive cultures, 25 cultures (35.2%) were polymicrobial, containing two or more distinct pathogens. Of these 71 patients, 65 received susceptibility-guided antimicrobial therapy before stent removal, whereas six did not receive targeted treatment because Staphylococcus epidermidis was the sole organism detected. In the overall culture‑guided group, 65 patients received culture‑guided, susceptibility‑aligned therapy and 35 received single‑shot fosfomycin.

**Table 2:** Pathogen spectrum in pre-stent-removal urine cultures (n = 71). Percentages refer to 71 positive cultures; totals exceed 100% due to polymicrobial growth

Pathogen	Number ofisolates (n)	Percentage ofpositive cultures (%)
Common uropathogens		
Escherichia coli	12	16.9
Klebsiella pneumoniae	7	9.9
Klebsiella oxytoca	3	4.2
Citrobacter freundii	1	1.4
Serratia rubidaea	1	1.4
Total:	24	33.8
Likely biofilm-associated pathogens		
Enterococcus faecalis	4	5.6
Candida albicans	22	31.0
Candida krusei	1	1.4
Enterococcus faecium	9	12.7
Staphylococcus aureus	1	1.4
Enterococcus spp. (unspecified)	4	5.6
Total:	41	57.7
Likely contamination		
Staphylococcus epidermidis	22	31.0
Staphylococcus haemolyticus	9	12.7
Staphylococcus hominis	3	4.2
Streptococcus oralis/mitis/gallolyticus	2	2.8
Total:	36	50.7

Mean treatment duration was 6.5 days in the culture-guided group and 10.3 days in the empirical nitrofurantoin group.

### Early infectious complications after ureteral stent removal

**Fig. 2:**
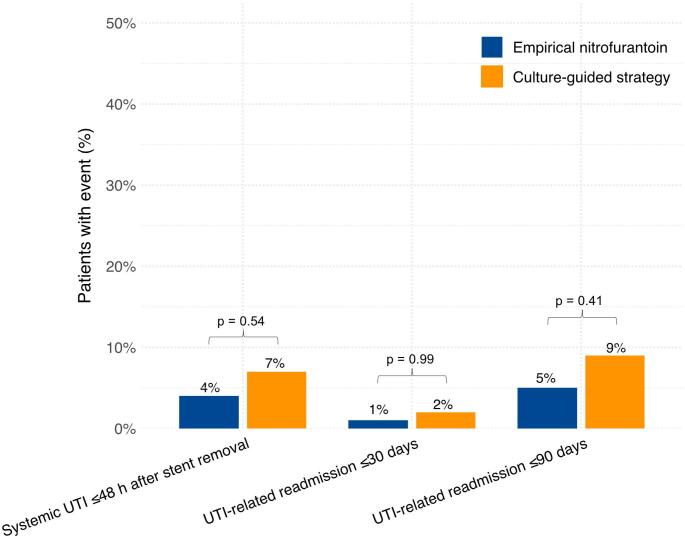
Infection-related outcomes by antimicrobial strategy for ureteral stent removal after radical cystectomy. Bar chart showing the proportion of patients with early systemic urinary tract infection (UTI) ≤ 48 h after ureteral stent removal and UTI-related readmissions within 30 and 90 days in the empirical nitrofurantoin versus culture-guided group

Early systemic UTIs within 48 h after ureteral stent removal occurred in 4% of patients in the empirical nitrofurantoin group (4/100) and 7% in the culture-guided group (7/100; OR 0.56, 95% CI 0.12-2.27; *p* = 0.54; Fig. 2). Notably, all patients with early systemic UTI in the culture-guided group (7/7, 100%) had a positive pre-removal urine culture with significant growth and were receiving susceptibility-guided antibiotic therapy at the time of stent removal. The identified pathogens among these seven patients were Candida albicans (*n* = 3), Enterococcus faecalis (*n* = 2), coagulase-negative staphylococci (*n* = 1), and Escherichia coli (*n* = 1). Antimicrobial regimens administered for ureteral stent removal according to susceptibility included fluconazole (*n* = 3), amoxicillin/clavulanic acid (*n* = 2), piperacillin/tazobactam (*n* = 1) and cotrimoxazole (*n* = 1). Rates of early systemic UTI ≤ 48 h after stent removal did not differ by UD type (*p* = 0.53). Changes in inflammatory markers before and after ureteral stent removal were small and did not differ between groups. The mean change in CRP (ΔCRP) was 49.3 mg/L (SD 69.9) in the empirical group and 48.2 mg/L (SD 61.1) in the culture‑guided group (*p* = 0.25); the mean change in leukocyte count (ΔLeuk) was 0.83 × 10⁹/L (SD 6.2) versus 0.42 × 10⁹/L (SD 3.46; *p* = 0.64). When stratified according to the occurrence of early systemic UTI, the increase in leukocyte count (ΔLeuk) was significantly higher in patients who developed early systemic UTI than in those without systemic UTI (2.65 vs. 0.51 × 10⁹/L; *p* = 0.048), whereas the corresponding difference in ΔCRP did not reach statistical significance (70.7 vs. 47.4 mg/L; *p* = 0.10).

### Hydronephrosis and reinterventions

New‑onset hydronephrosis after ureteral stent removal was observed in 27% of patients in the culture‑guided group (27/100) and 14% in the empirical group (14/100; *p* = 0.035). Only 2 of the 41 patients (5%) who developed hydronephrosis within 24 h after stent removal also presented with early systemic UTI. Reinterventions related to newly developed hydronephrosis (ureteral re‑stenting or percutaneous nephrostomy) were required in 9/27 patients in the culture‑guided group and 4/14 patients in the empirical group, with no statistically significant difference between regimens (*p* = 0.25).

### Infection‑related readmissions

Within 30 days after RC, UTI‑related readmission occurred in 1% of patients in the empirical nitrofurantoin group (1/100) and 2% in the culture‑guided group (2/100; OR 2.01, 95% CI 0.10-120.30; *p* = 0.99; Fig. 2). At 90 days, UTI‑related readmissions were observed in 5% (5/100) versus 9% (9/100) of patients in the empirical and culture‑guided group, respectively (OR 1.87, 95% CI 0.54-7.40; *p* = 0.41; Fig. 2). Overall, infection‑related readmissions did not differ significantly between antimicrobial strategies.

## Discussion

Infectious complications after ureteral stent removal represent one of the most frequent postoperative complications following RC, underscoring the clinical relevance of optimizing peri-interventional management. This study provides a direct comparison of empirical and culture-guided antimicrobial strategies at the time of ureteral stent removal. We hypothesized that culture-guided antimicrobial therapy would reduce early post-stent-removal infectious complications. However, both antimicrobial strategies were associated with similar rates of early systemic UTIs after stent removal. A key practical limitation of routine pre-removal culturing in stented RC patients is that positive cultures are common and often dominated by biofilm-associated or colonizing organisms, which may not be causally related to post-removal systemic infections. As a consequence, culture-guided pathways can trigger antimicrobial escalation without improving outcomes.

In the culture‑guided group, a substantial proportion of significant growth consisted of non‑classical uropathogens, predominantly Candida species, coagulase‑negative staphylococci and enterococci, highlighting the distinct colonization patterns associated with ureteral stents and UDs [10]. This microbiological pattern, together with the lack of a significant reduction in early systemic UTIs ≤ 48 h after stent removal despite targeted therapy, suggests that routine pre‑removal urine culture‑based adjustment of antibiotics, at least as implemented in our setting, may not be warranted. Pre-removal cultures frequently identify organisms that are unlikely to cause clinically relevant systemic UTIs, yet the presence of significant growth may prompt escalation of antimicrobial therapy and thereby promote unnecessary treatment of colonizing or biofilm-associated flora and contribute to antimicrobial resistance. Moreover, even when pre-removal urine cultures are obtained, they may not reflect the microbiology of the stent itself: Tulone et al. demonstrated that ureteral stents are colonized far more frequently than concurrent urine cultures suggest, with consistently low concordance between stent and renal pelvis urine cultures [11]. This is clinically relevant in the context of stent removal, as biofilm-associated organisms on the device surface may be mobilized at the time of extraction and drive post-procedural infectious events. These findings further question the rationale of routine culture-guided antimicrobial escalation based on pre-removal urine cultures in stented RC patients. In the absence of clear criteria defining which pathogens and colony count thresholds should prompt antimicrobial treatment in stented RC patients, clinicians may tend to treat microbiologically significant growth in this high-risk setting, including organisms that are more likely to represent colonization or contamination. This issue is particularly relevant in cases of candiduria. Although guidelines recommend antifungal therapy before urological manipulation in high-risk settings, candiduria alone usually reflects colonization rather than invasive infection [12]. Among the seven patients who developed systemic UTI in the culture‑guided group, three had candiduria and received susceptibility-guided fluconazole, yet still developed symptoms. This pattern suggests that the detected candiduria was unlikely the true driver of these events and illustrates how a treatment approach to culture results in stent patients can lead to antimicrobial agent use without clear clinical benefit.

Empirical nitrofurantoin throughout the stenting period was not associated with an increased incidence of early post-stent-removal systemic UTIs in our cohort, as compared with selective, culture-guided antimicrobial management, despite the latter being tailored to pathogens identified in pre-removal urine cultures. This finding suggests that nitrofurantoin may be a pragmatic empirical option. However, empirical nitrofurantoin administration resulted in a significantly longer cumulative antibiotic exposure (10.3 vs. 6.5 days; *p* < 0.001). Concerns regarding this extended exposure may be mitigated by nitrofurantoin’s low propensity for selecting resistance and its favorable short-term safety profile [13–15]. While common adverse effects such as nausea, vomiting or loss of appetite are generally mild, severe toxicities including interstitial pneumonitis or hepatitis are typically associated with prolonged nitrofurantoin exposure over several months (> 6 months) [16, 17]. Thus, an empirical course of approximately 10 days appears clinically acceptable. However, from an antimicrobial stewardship (AMS) perspective, cumulative antibiotic exposure remains a relevant concern. In this context, evidence from the broader RC literature supports more restrictive approaches: a recent randomized trial by Thurnheer et al. demonstrated noninferiority of 24-hour perioperative antibiotic prophylaxis compared with extended prophylaxis, while adverse events occurred exclusively in the extended group [18]. Viewed against this background, the extended antibiotic exposure observed in both groups of our cohort warrants critical reflection and further supports the need to prospectively evaluate shorter antimicrobial strategies in this setting.

In contrast, although patients in the culture-guided group received antibiotics for a shorter duration, this advantage may be offset by the agents used. A substantial proportion of patients received broad-spectrum antibiotics such as piperacillin-tazobactam or reserve agents such as linezolid, which, despite the absence of evidence for a clinically meaningful benefit, is problematic from an AMS perspective. Escalation to such broad-spectrum agents is known to promote multidrug-resistant organisms and carries a substantial risk of adverse drug events [19, 20].

Biomarker monitoring with CRP did not meaningfully differentiate between patients who did and did not develop early systemic UTI, whereas an early increase in leukocyte count was associated with early systemic UTI episodes after stent removal. Nevertheless, because routine laboratory measurements were obtained approximately 24 h after stent removal, delayed CRP peaks may have gone undetected. While markedly elevated CRP on postoperative day 4 (e.g., > 150 mg/L) has been proposed as a potential early warning sign for major complications after RC, its predictive value for severe sepsis or critical illness in surgical patients remains limited [21, 22]. Overall, peri‑removal laboratory surveillance around the time of ureteral stent removal appears to be clinically meaningful, as early leukocytosis may indicate an increased risk of a complicated course. One potential alternative to routine prophylaxis could therefore be a response-guided strategy, in which antimicrobial therapy is initiated only in patients who demonstrate an early leukocyte rise. In contrast, CRP appears less useful for short‑term risk stratification in this setting.

The higher incidence of hydronephrosis after stent removal in the culture-guided group was statistically significant, yet most cases were mild, transient and rarely associated with systemic UTIs or the need for reintervention. Although ureteritis secondary to UTI can contribute to transient obstruction, this mechanism was unlikely to be the predominant cause in our cohort. The absence of a consistent association between hydronephrosis and infectious events supports the interpretation that these findings largely reflect physiological postoperative changes rather than clinically meaningful ureteral inflammation or ascending infection with obstruction.

The pathophysiological mechanism leading to infectious complications after ureteral stent removal in RC patients remains incompletely understood. During the early postoperative healing phase, vulnerable urothelial surfaces or subtle anastomotic narrowing may predispose to mucosal microtrauma during stent removal, potentially facilitating bacterial passage. Additionally, even minimal urine extravasation in the anastomotic area could provoke systemic inflammatory responses. Transient elevations in intrarenal pressure, which are plausible after stent removal, can induce pyelovenous backflow, enabling the translocation of fluid and bacteria into the venous system [23].

Lastly, it is worth noting that two randomized controlled trials and a recent systematic review and meta-analysis reported higher odds of major postoperative complications in stented versus unstented RC patients, without clinically relevant differences in readmission or anastomotic outcomes, thereby supporting stent omission during RC as a reasonable option [24–28]. These findings position the present comparative cohort study on antimicrobial strategies at the time of stent removal as a complementary contribution to the broader discussion on whether ureteral stents should be used routinely during RC at all.

Based on our findings, empirical nitrofurantoin may represent a pragmatic and stewardship-aligned strategy for ureteral stent removal after RC. Nitrofurantoin is simple to administer orally, associated with relatively low rates of resistance development and did not demonstrate worse early infection-related outcomes compared with the culture-guided approach in our cohort. Nevertheless, given the longer duration of administration, minimizing cumulative antibiotic exposure remains an important stewardship goal. Even more restrictive strategies, including shorter courses of nitrofurantoin, alternative short-duration regimens, or the omission of procedure-specific prophylaxis altogether, may warrant prospective evaluation. Further clarification will depend on larger prospective datasets, including results from the ongoing REINFORCE trial, which compares a culture-guided approach with empirical pivmecillinam for stent removal after RC, using UTI-related readmission at 90 days as the primary endpoint [29]. Its results will provide high-level evidence on whether an individualized culture-guided approach offers clinically meaningful advantages over empirical prophylaxis.

This study is not free of limitations: First, its retrospective design entails an inherent risk of selection bias. Second, the pre-post-design inherently introduces a risk of secular confounding, as other changes in perioperative care (e.g. surgeon experience, nursing protocols, organizational factors) over time may have influenced outcomes independently of the antimicrobial strategy for stent removal. Furthermore, as with any retrospective study, the possibility of unmeasured confounding cannot be excluded. Variables not routinely captured in the EHR may have differentially influenced infectious outcomes between groups. Additionally, factors besides stent removal may lead to systemic UTI episodes and infectious complications following RC. Although we focused on early systemic UTIs occurring within 48 h after stent removal to mitigate this limitation, competing causes or triggers of systemic UTI cannot be excluded. Moreover, given the clinically based EAU definition of systemic UTI, some events may have reflected non-urinary-tract-related postoperative complications with overlapping systemic features. Finally, the overall number of early systemic UTIs within 48 h after stent removal was low, and the study was not powered to detect small between-group differences.

Despite these constraints, our findings provide preliminary evidence that a clinically meaningful advantage of a culture-guided antimicrobial strategy over empirical nitrofurantoin for stent removal in RC patients is questionable and that major differences between the two approaches are unlikely, thereby supporting further prospective evaluation.

## Conclusion

Our findings provide preliminary evidence that empirical nitrofurantoin and a culture-guided antimicrobial approach are associated with similar rates of early systemic UTIs after ureteral stent removal in RC patients. Accordingly, routine culture-guided adjustment of antibiotics for stent removal may add limited clinical value while promoting unnecessary antimicrobial escalation. Larger-scale, prospective studies are required to generate robust evidence and define optimal, procedure-specific antimicrobial strategies for this high-risk patient population. 

## Electronic Supplementary Material

Below is the link to the electronic supplementary material.

Supplementary Material 1

## Data Availability

No datasets were generated or analysed during the current study.
