# Supplementary material for: Antimicrobial strategies for ureteral stent removal after radical cystectomy: a comparative cohort study
Source: Eur J Clin Microbiol Infect Dis. 2026 Apr 30;45(8):2495–504. doi: 10.1007/s10096-026-05514-4 (PMC13428704; doi:10.1007/s10096-026-05514-4)
Supplement: Supplementary file 1 — Supplementary Material 1 [file 10096_2026_5514_MOESM1_ESM.docx]

**Supplementary Table 1:** STROBE checklist for control, case-control, and cross-sectional studies. (https://www.strobe-statement.org/)

|  | Item No. | Recommendation | Page  No. | Relevant text from manuscript |
| --- | --- | --- | --- | --- |
| **Title and abstract** | 1 | (*a*) Indicate the study’s design with a commonly used term in the title or the abstract | 2 | Abstract |
|  |  | (*b*) Provide in the abstract an informative and balanced summary of what was done and what was found | 2 | Abstract: Methods, Key results and limitations |
| Introduction | | | |  |
| Background/rationale | 2 | Explain the scientific background and rationale for the investigation being reported | 3 | Introduction, Ln-65-89 |
| Objectives | 3 | State specific objectives, including any prespecified hypotheses | 3 | Introduction, Ln. 90-97 |
| Methods | | | |  |
| Study design | 4 | Present key elements of study design early in the paper | 4 | Methods, Paragraph 2.1 |
| Setting | 5 | Describe the setting, locations, and relevant dates, including periods of recruitment, exposure, follow-up, and data collection | 4 | Methods, Paragraph 2.1, Paragraph 2.3 |
| Participants | 6 | (*a*) *Cohort study*—Give the eligibility criteria, and the sources and methods of selection of participants. Describe methods of follow-up  *Case-control study*—Give the eligibility criteria, and the sources and methods of case ascertainment and control selection. Give the rationale for the choice of cases and controls  *Cross-sectional study*—Give the eligibility criteria, and the sources and methods of selection of participants | 4 | Methods, Paragraph 2.1 |
|  |  | (*b*) *Cohort study*—For matched studies, give matching criteria and number of exposed and unexposed  *Case-control study*—For matched studies, give matching criteria and the number of controls per case | n.a. | n.a. |
| Variables | 7 | Clearly define all outcomes, exposures, predictors, potential confounders, and effect modifiers. Give diagnostic criteria, if applicable | 5 | Methods, Paragraph 2.2 |
| Data sources/ measurement | 8* | For each variable of interest, give sources of data and details of methods of assessment (measurement). Describe comparability of assessment methods if there is more than one group | 5 | Methods, Paragraph 2.4 |
| Bias | 9 | Describe any efforts to address potential sources of bias | 4  23 | Methods, Ln. 114-118  Results, Ln. 338-340 |
| Study size | 10 | Explain how the study size was arrived at | 4 | Methods, Ln. 114-118 |

Continued on next page

| Quantitative variables | 11 | Explain how quantitative variables were handled in the analyses. If applicable, describe which groupings were chosen and why | 5 | Methods, Paragraph 2.4 |
| --- | --- | --- | --- | --- |
| Statistical methods | 12 | (*a*) Describe all statistical methods, including those used to control for confounding | 5 | Methods, Paragraph 2.4 |
|  |  | (*b*) Describe any methods used to examine subgroups and interactions | 5 | Methods, Paragraph 2.4 |
|  |  | (*c*) Explain how missing data were addressed | n.a. | n.a. |
|  |  | (*d*) *Cohort study*—If applicable, explain how loss to follow-up was addressed  *Case-control study*—If applicable, explain how matching of cases and controls was addressed  *Cross-sectional study*—If applicable, describe analytical methods taking account of sampling strategy | 5 | Methods, Paragraph 2.4 |
|  |  | (*e*) Describe any sensitivity analyses | 5 | Methods, Paragraph 2.4 |
| Results | | | | |
| Participants | 13* | (a) Report numbers of individuals at each stage of study—eg numbers potentially eligible, examined for eligibility, confirmed eligible, included in the study, completing follow-up, and analysed | 6 | Results, Ln. 160-165 |
|  |  | (b) Give reasons for non-participation at each stage | 5 | Methods, Paragraph 2.4 |
|  |  | (c) Consider use of a flow diagram | n.a. | n.a. |
| Descriptive data | 14* | (a) Give characteristics of study participants (eg demographic, clinical, social) and information on exposures and potential confounders | 6 | Results, Ln. 160-165 |
|  |  | (b) Indicate number of participants with missing data for each variable of interest | n.a. | n.a. |
|  |  | (c) *Cohort study*—Summarise follow-up time (eg, average and total amount) | n.a. | n.a. |
| Outcome data | 15* | *Cohort study*—Report numbers of outcome events or summary measures over time | 7-9 | Results, Paragraph 3.1-3.3 |
|  |  | *Case-control study—*Report numbers in each exposure category, or summary measures of exposure |  |  |
|  |  | *Cross-sectional study—*Report numbers of outcome events or summary measures |  |  |
| Main results | 16 | (*a*) Give unadjusted estimates and, if applicable, confounder-adjusted estimates and their precision (eg, 95% confidence interval). Make clear which confounders were adjusted for and why they were included | 6-8 | Results |
|  |  | (*b*) Report category boundaries when continuous variables were categorized | n.a. | n.a. |
|  |  | (*c*) If relevant, consider translating estimates of relative risk into absolute risk for a meaningful time period | n.a. | n.a. |

Continued on next page

| Other analyses | 17 | Report other analyses done—eg analyses of subgroups and interactions, and sensitivity analyses | n.a. | n.a. |
| --- | --- | --- | --- | --- |
| Discussion | | | | |
| Key results | 18 | Summarise key results with reference to study objectives | 9 | Discussion, Ln. 218-225 |
| Limitations | 19 | Discuss limitations of the study, taking into account sources of potential bias or imprecision. Discuss both direction and magnitude of any potential bias | 12 | Discussion, Ln. 337-346 |
| Interpretation | 20 | Give a cautious overall interpretation of results considering objectives, limitations, multiplicity of analyses, results from similar studies, and other relevant evidence | 9-12 | Discussion |
| Generalisability | 21 | Discuss the generalisability (external validity) of the study results | 12  12 | Discussion, Ln. 325-335  Conclusion |
| Other information | |  | | |
| Funding | 22 | Give the source of funding and the role of the funders for the present study and, if applicable, for the original study on which the present article is based | 16 | Statements and Declarations |

*Give information separately for cases and controls in case-control studies and, if applicable, for exposed and unexposed groups in cohort and cross-sectional studies.
